# Understanding Technology Adoption, Troubleshooting, and Telehealth Among the Lifestyle Leaders

**DOI:** 10.1093/geroni/igab046.1071

**Published:** 2021-12-17

**Authors:** John Rudnik

**Affiliations:** University of Michigan School of Information

## Abstract

The COVID-19 pandemic has prompted widespread adoption of and greater reliance on digital technologies across the generations. In interviews and focus groups, the Lifestyle Leaders reported increased use of teleconferencing services, especially for virtual programming, socializing, and telehealth. Approximately 61% of Lifestyle Leaders had attended between one and five virtual or phone-based healthcare appointments since March 2020. Beliefs about telehealth across interviews varied: appointments were convenient but lacked the “in-person feedback and understanding” of an in-person visit. Half of the Lifestyle Leaders (50%) agreed with the statement, “Telehealth is the future of personalized medicine.” This presentation will also report on the Lifestyle Leaders’ challenges associated with using technology during the pandemic. These included concerns about: having devices able to keep up with the demands of the pandemic; using technology-enabled grocery services; and troubleshooting technology. Many were concerned about their growing dependence on technology because of the pandemic.

